# The hidden cost of health misinformation: distrust, treatment refusal, and the burden on healthcare professionals

**DOI:** 10.3389/fmed.2026.1842755

**Published:** 2026-07-20

**Authors:** Daniel Catalan-Matamoros, Mariola Moreno, Carmen Peñafiel-Saiz

**Affiliations:** 1 MediaLab, Department of Communication and Media Studies, Madrid University Carlos III, Madrid, Spain; 2 Institute for Culture and Society, University of Navarra, Pamplona, Spain; 3 Department of Journalism, University of the Basque Country, Bilbao, Spain

**Keywords:** health literacy, health misinformation, infodemic, patient trust, therapeutic alliance

## Abstract

**Background:**

Health misinformation has become a persistent challenge in contemporary clinical practice, shaping patient beliefs, weakening trust in healthcare professionals, and contributing to the refusal of evidence-based treatments. In the context of the growing influence of digital platforms, frontline health professionals increasingly face consultations in which misinformation must be addressed alongside clinical care.

**Methods:**

A qualitative descriptive design based on semi-structured interviews was administered through an online open-ended questionnaire. Healthcare professionals working in Spain participated in the study. Data were analyzed using inductive thematic analysis to identify recurring patterns related to health misinformation, patient trust, treatment refusal, and professional communication strategies.

**Results:**

The analysis revealed five themes: (1) information sources and misinformation exposure, (2) erosion of trust and clinical friction, (3) patient refusal of evidence-based treatment, (4) the healthcare professional as a debunker, and (5) the need for media literacy and professional training. Participants consistently reported that patients rely heavily on social media, search engines, and non-expert sources, often arriving to consultations with pre-formed beliefs that contradict medical advice. They described misinformation as a source of distrust, emotional exhaustion, and poorer communication in the therapeutic relationship.

**Discussion:**

The findings suggest that misinformation is not merely an online phenomenon but a concrete clinical problem that directly affects trust, communication, and treatment adherence. Healthcare professionals perceive themselves as having an increasingly important educational role, yet many lack formal preparation to respond effectively to misinformation in practice. These results support the integration of health communication and media literacy into professional training.

## 1 Introduction

In the contemporary digital era, the dissemination of health information has reached unprecedented speed and ubiquity. However, this democratization of access to knowledge coexists with an information ecosystem increasingly polluted by the rapid spread of misinformation, defined as false or misleading information shared without the intention to mislead, and disinformation, defined as false or misleading information intentionally spread to mislead (1). Recent global health crises, exacerbated by the COVID-19 pandemic and the so-called “infodemic” (2, 3), have demonstrated that continuous exposure to anti-scientific narratives is not harmless. There is robust empirical evidence correlating the consumption of inaccurate information with adverse public health outcomes, including a significant increase in vaccine hesitancy and the adoption of treatments lacking empirical evidence (4).

The proliferation of these narratives is facilitated by digital platforms that operate through algorithms designed to maximize attention (2). These algorithms create “echo chambers” that isolate individuals from evidence-based information and exploit psychological and sociocultural vulnerabilities. In our study, the term “echo chambers” is used descriptively to refer to environments in which users are primarily exposed to viewpoints that reinforce their existing beliefs, while acknowledging that the concept remains debated in the literature and may overlap with effects of personalization and recommendation systems (3). Related to this discussion is the concept of “data voids,” which refers to search queries that return little or no relevant information and may be exploited to amplify misleading content (4).

As citizens increasingly turn to social media and other types of media for health information, they may encounter content that is not always critically assessed or verified (5), the figure of the “wellness influencer” and fast-paced content formats on platforms like TikTok are rapidly replacing traditional medical sources (1, 5). This paradigm shift not only poses a profound challenge to digital media literacy but also drives a concerning erosion of public trust in health authorities and institutional science (6).

Despite systemic efforts to mitigate these trends at a macro level, one of the most profound and least empirically explored impacts occurs on the frontline of healthcare delivery: the doctor-patient relationship. The therapeutic alliance is historically and clinically founded on mutual trust. Today, however, healthcare professionals face a new paradigm of care. They are forced to invest considerable clinical time and effort not only in diagnosing and treating, but also acting as misinformation correctors (debunking), dealing with patients who come to the consultation with deeply rooted false beliefs (7).

This dynamic generates notable friction in clinical communication (8) and, even more alarmingly, directly influences the patient's decision-making process, occasionally leading to the explicit refusal of preventive or life-saving therapies based on viral conspiracy theories. While prior research has largely examined health misinformation through its prevalence and diffusion on digital platforms and its association with vaccine hesitancy, risk perceptions, and institutional distrust at the population level, most of this work has focused on online content patterns and general public attitudes (9–12). In contrast, empirical studies that explore how frontline healthcare professionals experience these dynamics in consultations, particularly in relation to the therapeutic alliance, clinical friction, and treatment refusal, remain comparatively scarce. So there remains a substantial gap in understanding how healthcare workers directly experience and manage these barriers in their daily clinical practice (7). By focusing on healthcare professionals' narratives, this study contributes to bridging this gap and offers contextualized evidence on the clinical implications of misinformation and the perceived need for communication and media literacy training.

Therefore, the aim of this study is to explore and describe the impact of health misinformation on daily clinical practice, specifically evaluating how exposure to false health content affects the therapeutic relationship and influences refusal of evidence-based treatments.

## 2 Method

This section describes the methodological approach employed in this study, including study design and ethical considerations (2.1), participant recruitment and characteristics (2.2), data collection procedures (2.3), and data analysis techniques (2.4).

### 2.1 Study design, ethics, and study sample

This study employed a qualitative descriptive design based on semi-structured interviews, administered as an online questionnaire with open-ended questions. The study was conducted in accordance with the ethical guidelines of the Declaration of Helsinki and was approved by the Ethics Committee of Madrid University Carlos III (Ref. CEI-25-02-COMSALUD). Informed consent was obtained from all participants prior to data collection, ensuring anonymity, confidentiality, and the exclusive use of the information for academic research purposes at all times.

The instrument was designed to ensure neutrality and minimize bias, supporting the validity of the findings.

### 2.2 Participants

The final sample consisted of 26 healthcare professionals practicing in Spain. Participant selection was carried out using convenience and snowball sampling techniques. Participants were eligible if they were healthcare professionals currently practicing in Spain, had direct patient contact in their daily work, and had at least 1 year of clinical experience. Initial participants were contacted through professional networks and they were invited to share the link with colleagues.

The age of the participants ranged from 28 to 75 years, with a mean age of 50 years. Regarding gender distribution, the sample was pre-dominantly female, representing 76.9% (*n* = 20), compared to 23.1% male (*n* = 6).

**Figure 1:**
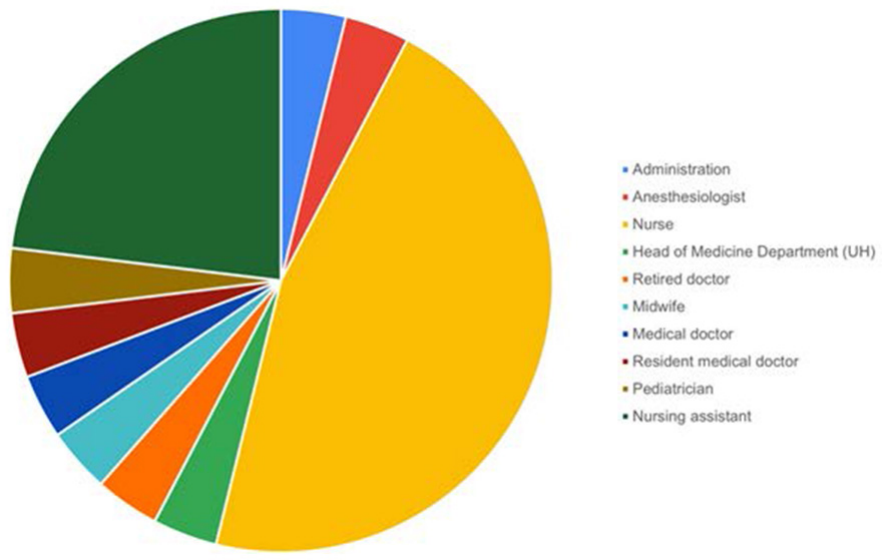
Professional profiles of participants.

All participants reported having academic training in the field of Health Sciences. The professional profiles were varied to obtain a multidisciplinary perspective and included: nurses (*n* = 12), medical doctors from various specialties (*n* = 5), nursing assistants (*n* = 6), midwives (*n* = 1), health management staff (*n* = 1), and a retired healthcare professional (*n* = 1) (see Figure 1). Regarding the highest educational level achieved, 61.5% held a Bachelor's or Master's degree (*n* = 16), 26.9% had vocational or secondary education (*n* = 7), and 11.5% held a Ph.D. (*n* = 3).

The questionnaire was distributed through a personalized invitation process to a pre-selected sample of participants. All individuals who were contacted agreed to participate and completed the interview in full. Consequently, no partially completed or abandoned questionnaires were recorded.

### 2.3 Data collection

Data collection was conducted between May and June 2025 using a self-administered online form. The instrument consisted of two main blocks:

Sociodemographic data: information was collected on age, gender, educational level, field of study, current profession, place of residence, and political ideology. These sociodemographic data were included as a descriptive sociodemographic variable to characterize the sample and provide contextual information. They were not used as a primary outcome or analytic category in the thematic analysis.

Semi-structured interview: this section included 11 open-ended questions designed to elicit descriptive responses. These were divided into questions about the professionals' own information consumption habits (i.e., primary information sources, level of trust in media, perception of the COVID-19 infodemic) and direct clinical questions (i.e., how misinformation affects their clinical work, recent cases of patients refusing evidence-based treatments due to conspiracy beliefs, and communication strategies used during consultations to debunk myths).

### 2.4 Data analysis

A mixed approach was adopted for data analysis. First, sociodemographic data were analyzed using descriptive statistics (frequencies, percentages, and means) to define the sample profile. Second, for the qualitative analysis of the responses, an inductive thematic analysis approach was utilized by two researchers, experts in Health Communication, based on the method by Braun and Clarke (13). All qualitative responses were analyzed collectively once data collection had been completed. Given the descriptive nature of the study and the open-ended format of the responses, no post-collection data cleaning procedures were applied beyond eligibility verification; likewise, for the quantitative data, no inferential tests or effect sizes were calculated and *p*-values are not reported. Responses were analyzed as originally submitted.

The participants‘ textual responses were read repeatedly to achieve familiarization with the data. Subsequently, systematic manual coding was performed through a process of discussion and consensus between the two researchers, allowing categories and themes to emerge directly from the data through an iterative process of analysis and refinement. Five main themes emerged from the analysis: (1) The landscape of misinformation, sources and content; (2) The erosion of trust and its impact on clinical work; (3) The health professional as a “Debunker”: strategies and self-perception; (4) The perceived role of the healthcare professional in the fight against pseudoscience; and (5) Professional training. Manual coding was intentionally selected over automated qualitative analysis software because the study aimed to capture contextual nuances, implicit meanings, and interpretative dimensions within healthcare professionals' narratives. Given the exploratory and inductive nature of the study, researcher-led coding was considered more appropriate to preserve semantic depth and contextual interpretation, which may be insufficiently captured through automated coding procedures alone.

The analysis focused on identifying recurring patterns in the healthcare workers' discourse regarding the clinical friction generated by misinformation, communication barriers in the therapeutic relationship, and the empirical mechanisms professionals use on a daily basis to debunk false health beliefs.

## 3 Results

The inductive thematic analysis of the responses provided by the 26 healthcare professionals revealed five major themes. These themes illustrate the trajectory of misinformation from digital platforms to the consultation room, highlighting its tangible effects on patient trust and medical decision-making.

### 3.1 Theme 1: the landscape of misinformation, sources and content

#### 3.1.1 Subtheme 1.1: the primacy of social media and “Dr. Google”

Professionals reported that patients frequently arrive at consultations with pre-existing beliefs shaped by online content. Rather than relying on professional advice, patients often base their opinions on unverified sources, which creates tension during consultations. The frustration with unverified online information was a recurring sentiment. One nurse noted that patients “believe more what they see or hear than what professionals tell them in many cases” [Nurse, 35 years old]. Another participant described the dynamic as frustrating, stating, “they trust Google more than medical information” [Nurse, 57 years old]. The role of social media was repeatedly emphasized as a source of “information not contrasted” [Nurse, 53 years old] and a space where “pseudoscience” flourishes.

#### 3.1.2 Subtheme 1.2: pervasive and recurrent misinformation topics

Three main categories of misinformation were identified:

- Vaccines: this was the most frequently mentioned topic. Professionals cited patients refusing vaccines for COVID-19, influenza, and other preventable diseases. One participant gave an example of patients who were “convinced that the COVID vaccine had caused them irreparable damage (sterilization, for example)” [Nurse, 45 years old].
- Antibiotics and treatments: several professionals mentioned patients demanding or refusing treatments based on misconceptions. One participant cited “vaccinations and antibiotics” as common points of contention [Nurse, 34 years old].
- Nutrition and pseudoscience: some professionals highlighted misinformation related to diet and health trends, particularly in weight-loss contexts, although these accounts were more descriptive than quote-based.
- COVID-19 conspiracy theories: the pandemic was described as a turning point, with an increase in misinformation. One nurse reported frequently encountering misinformation during the COVID-19 pandemic, especially misconceptions regarding vaccine side effects that were inconsistent with clinical evidence [Nurse, 45 years old], while another noted “questions related to my profession in doulas with economic or ideological interests behind them” [Midwife, 49 years old].

### 3.2 Theme 2: the erosion of trust and its impact on clinical work

The participants consistently reported that misinformation directly and negatively impacts the therapeutic relationship, creating friction, consuming time, and fostering distrust.

#### 3.2.1 Subtheme 2.1: strained therapeutic alliance

The need to constantly correct misinformation was perceived as emotionally and professionally demanding. This constant need to correct misinformation was described as “exhausting” [Nurse, 47 years old] and a source of significant friction. A physician noted that the infodemic “distorts the doctor-patient relationship a lot, as it generates distrust and countertransference” [Medical doctor, 75 years old]. Another nurse elaborated that the arrival of misinformed patients creates an “impossibility of doing [their] job well” as their clinical decisions are constantly questioned [Nurse, 50 years old]. A resident physician described the consequence of this dynamic: “misinformation makes the patient suspicious and distrustful regarding the decisions you make, leading to poor communication with them” [Medical doctor, 28 years old].

#### 3.2.2 Subtheme 2.2: patient refusal of evidence-based treatments

A significant number of participants reported encountering patients who directly refused treatments based on misinformation. This ranged from rejecting vaccines to refusing anesthesia. One professional recalled a patient who refused a surgical intervention because of fears and misinformation about not waking up after anesthesia [Nurse, 50 years old]. In this case, the professional identified that the patient's refusal was not rooted in common procedural anxiety, but was instead exacerbated by “conspiracy theories” and unverified anecdotal accounts found online that portray medical interventions as intentionally harmful or inherently fatal. This reflects a broader trend observed in our results where patients, influenced by digital “echo chambers”, develop a profound distrust of institutional science and evidence-based protocols, leading to the perception of standard clinical risks as insurmountable threats rather than manageable variables. Supporting this observation, another participant highlighted cases of patients rejecting “conventional treatment for preferring homeopathic treatment” [Nurse, 53 years old], while a retired professional [65 years old] concisely noted “frequent rejections of vaccines” as a recurring challenge in their experience.

### 3.3 Theme 3: the health professional as a “debunker”, strategies and self-perception

Participants revealed a range of strategies to combat misinformation, demonstrating a reactive and often improvised approach, alongside a clear sense of the challenges involved.

#### 3.3.1 Subtheme 3.1: a triad of strategies: evidence, data, and respect

The most frequently cited strategies relied on presenting scientific evidence.

- Reasoning with data: a common approach was “reasoning with data and evidence” [Medical doctor, 65 years old], citing “meta-analyses” [Head of medical services, 75 years old] and “scientific articles” [Resident medical doctor, 28 years old].
- Relying on evidence-based knowledge: participants described their primary tool as “scientific evidence” [Nurse, 35 years old], “facts” [Nurse, 45 years old], or “tangible results” [Nurse, 41 years old].
- Diplomacy and respect: a more nuanced approach was described by a midwife who used “information, respect, non-imposition” [Midwife, 49 years old], highlighting the delicate nature of these conversations. Another professional emphasized “avoiding arrogance and dogmatism” [Head of medical services, 75 years old].

#### 3.3.2 Subtheme 3.2: perceived limitations and role conflict

Some participants expressed uncertainty or pessimism about their role as debunkers. One professional stated their role was “not to convince anyone” [Medical doctor, 43 years old], while another framed it as an impossible task, comparing “convincing an anti-vaxxer” to “convincing a Christian that God does not exist” [Head of medical services, 75 years old]. This underscores the psychological burden and perceived limitations of these clinical interactions.

### 3.4 Theme 4: the perceived role of the healthcare professional in the fight against pseudoscience

Despite the challenges, there was a strong consensus that healthcare professionals have a crucial role to play in countering misinformation, primarily through education and public engagement.

#### 3.4.1 Subtheme 4.1: education and public outreach

Participants strongly believed in their role as educators. One nurse argued that professionals “should carry out health education based on evidence” [Nurse, 45 years old]. Others called for a more proactive stance, suggesting that professionals should “be on social media and in the media giving truthful and evidence-based information” [Nurse, 35 years old] and that they have “a fundamental role” in “clarifying and teaching” [Nurse, 53 years old]. Another professional highlighted the importance of “educating society, inviting them” [Retired, 65 years old] to reliable sources.

#### 3.4.2 Subtheme 4.2: defenders of scientific evidence

The role was consistently defined as that of a defender of “scientific evidence”. One participant succinctly stated their role was to “defend scientific evidence” [Medical doctor, 65 years old], while another emphasized the importance of “responding with proven theories and arguments based on scientific evidence” [Nurse, 34 years old]. A third professional noted, “we are the ones who have to give or inform about truthful information” [Nurse, 49 years old].

### 3.5 Theme 5: professional training in media literacy

A notable finding was the lack of formal among healthcare professionals participants regarding the management of health misinformation, with many participants reporting that they relied primarily on self-directed learning and on-the-job clinical experience.

#### 3.5.1 Subtheme 5.1: the absence of formal training

When asked about specific training in health communication and media literacy, the majority of responses were negative, with many simply stating “No” [Nurse, 52 years old; Nurse, 34 years old; Nurse, 40 years old]. While some had received informal training or had “read up on the subject” [Nurse, 53 years old], a formalized approach was absent. One participant noted, “I have received training, I have looked for it on my own” [Nurse, 47 years old], highlighting the self-directed nature of their preparation.

#### 3.5.2 Subtheme 5.2: a call for proactive engagement

The data revealed a gap between the acknowledged importance of media literacy and the lack of structured preparation. The recognition that “the media only cause more disinformation” [Nurse, 45 years old] and that “the media influence a lot, a lot” [Nurse, 41 years old] coexists with a clear need for more effective tools, as professionals are currently managing this infodemic in the clinic with improvised strategies and significant personal effort.

#### 3.5.3 Subtheme 5.3: media literacy as a structural determinant

Finally, participants consistently identified media literacy as a key factor influencing patient behavior and public health outcomes. There was a shared perception that misinformation is not only a clinical issue but a systemic one.

As one participant summarized: “The problem is not just the misinformation itself, but that people don't have the tools to distinguish reliable information” [Nurse, 60 years old].

This reinforces the idea that addressing misinformation requires interventions beyond the clinical setting, integrating education, communication, and public health strategies.

## 4 Discussion

The present descriptive study provides insights into how health misinformation has transcended the digital ecosystem to become a tangible clinical barrier. The findings suggest that anti-scientific narratives and echo chambers not only alter public perception at a macro level but also have a direct, daily impact on the core of clinical practice: the therapeutic alliance.

The results evidence a concerning erosion of patient trust toward healthcare professionals, who are often viewed with suspicion when their recommendations contradict claims found on the internet. This clinical friction reported by the HCPs does not occur in a vacuum, it is framed within a broader context of healthcare system vulnerability. As Añel-Rodríguez and Rodríguez-Bilbao (14) point out, Primary Care is currently traversing a profound crisis characterized by citizen disaffection. In this scenario of institutional fragility, misinformation acts as a catalyst that deepens the disconnection between the patient and the evidence-based healthcare system.

This crisis of trust has provoked a displacement of “epistemic authority”. The physician is no longer the sole possessor of valid knowledge in the eyes of the patient. We are facing a contemporary paradox: citizens have access to an unprecedented amount of health-related data, but, as Gérvas and Pérez-Fernández (15) warn when analyzing health services, “more is not always better”. This overabundance of unfiltered information (infoxication) clouds the patient's clinical judgment and fosters a false sense of expertise.

The most alarming consequence of this dynamic is the direct refusal of consolidated medical interventions, a phenomenon our participants frequently observed regarding vaccination and antibiotic treatments. The abandonment of empirical therapies in favor of unsupported alternatives (such as homeopathy or miracle diets) generates real clinical risks. Furthermore, reversing these false beliefs once the patient has assimilated them poses a monumental cognitive challenge. As high-impact external literature points out, direct correction is often ineffective because misinformation is intrinsically “sticky” and difficult to eradicate from a patient's reasoning, even after being debunked with solid evidence (16).

Faced with this challenge, our study indicates that many frontline healthcare workers might experience what they describe as a form of ‘informational burnout'. Professionals assume the pedagogical burden of debunking myths daily, a task that directly clashes with the chronic lack of time in public system consultations. Healthcare providers are acutely aware that simply presenting statistical data or meta-analyses is insufficient to persuade patients who have been radicalized by algorithms (17). To combat this dynamic, the active promotion of health literacy across all spheres of community care becomes indispensable. Fostering this literacy requires the joint effort of various actors to empower patients and improve their critical decision-making (18).

However, for healthcare workers to assume this role of “media educators”, an urgent update in their academic training is required. Our results support the growing demand to integrate and strengthen communicative competencies within university curricula, as asserted by Gorricho-Genua et al. (19). Recent scientific evidence demonstrates that specific educational interventions aimed at improving the health communication skills of professionals generate positive, tangible effects when managing these types of informational crises at the community level (20). Without these competencies formalized in the curriculum, professionals will continue facing the infodemic equipped only with intuition and empirical diplomacy, thereby perpetuating the emotional toll documented in this work.

This study presents certain limitations inherent to its qualitative design. The sample size was modest and obtained through convenience and snowball sampling, with participants primarily practicing in Spain, and the overrepresentation of the female gender suggest that the findings should be interpreted within this specific sociocultural context. In addition, the use of self-reported narratives is subject to self-selection, social desirability, and recall biases, and the qualitative design does not allow for statistical generalization. The thematic analysis reflects the perspectives of participating healthcare professionals and the interpretive lens of the researchers rather than providing direct observational evidence of consultations. These limitations should be taken into account when interpreting the results and highlight the need for future studies with larger, more diverse samples and complementary methodological approaches.

The arrival of the “infodemic” in medical consultation rooms has transformed the paradigm of clinical care, forcing healthcare professionals to compete with digital algorithms to maintain their patients' trust. This study highlights that misinformation is no longer merely a theoretical public health issue, but a daily source of friction and an imminent risk to the individual patient. Our findings underscore the importance of institutional efforts to protect the therapeutic alliance, for example by not only with clinical guidelines but through a profound restructuring of consultation time, rigorous media literacy programs, and the systematic development of communicative competencies starting from university education.

## Data Availability

The raw data supporting the conclusions of this article will be made available by the authors, without undue reservation.
