# Supplementary material for: The hidden cost of health misinformation: distrust, treatment refusal, and the burden on healthcare professionals
Source: Front Med (Lausanne). 2026 Jul 20;13:1842755. doi: 10.3389/fmed.2026.1842755 (PMC13429649; doi:10.3389/fmed.2026.1842755)
Supplement: Supplementary file 1 [file Table_1.docx]

**Interview questionnaire**

**Sociodemographic Information**

1. What is your age?
2. Which gender do you identify with?
3. What is the highest level of education you have completed?
4. What is your current occupation or profession?
5. Where do you currently live? (City/town and province/state)
6. What is your political ideology? Please indicate a number where 1 means far left and 10 means far right.

**Health Information-Seeking and Consumption Habits**

1. What are your main sources of information on health-related topics?
2. How much trust do you place in health information circulated through digital and traditional media?
3. Have you recently identified cases of misinformation, pseudoscience, or conspiracy theories related to health? How did you recognize them?
4. In what ways do you think the COVID-19 pandemic has changed your perception and consumption of health information?
5. How important do you consider media literacy for identifying reliable health information?
6. Do you trust science and applications such as vaccines to promote human well-being?

**Healthcare Professionals’ Experiences in the Context of Disinformation**

1. How does misinformation or fake news affect your clinical practice or your relationship with patients?
2. Have you had patients who rejected treatments because of conspiracy theories? (Yes/No + brief example)
3. Have you received specific training in health communication and media literacy?
4. What role do you think healthcare professionals should play in combating pseudoscience?
5. What strategy do you use to convince anti-vaccine individuals?
